# Pilot data findings from the Gothenburg treatment for gaming disorder: a cognitive behavioral treatment manual

**DOI:** 10.3389/fpsyt.2023.1162492

**Published:** 2023-06-06

**Authors:** Annika Hofstedt, Mikael Mide, Elin Arvidson, Sofia Ljung, Jessica Mattiasson, Amanda Lindskog, Anna Söderpalm-Gordh

**Affiliations:** ^1^Institute of Neuroscience and Physiology, Sahlgrenska Academy, University of Gothenburg, Gothenburg, Sweden; ^2^Department of Addiction and Dependency, Sahlgrenska University Hospital, Gothenburg, Sweden

**Keywords:** adolescents, young adults, adults, treatment, CBT, gaming disorder, pilot

## Abstract

**Background:**

Gaming disorder (GD) is a new diagnosis included in the latest edition of the International Classification of Disease −11. Recently conducted international studies suggest a prevalence rate close to 2% for GD, highlighting the need for effective treatments for this patient population. Internationally there are few studies investigating effective treatments specifically designed for this condition. In this pilot study, we wanted to test a newly developed method, the Gothenburg Treatment for Gaming Disorder (GOT-TO-GO) manual; a 15-week cognitive behavioral therapy treatment for GD.

**Method:**

This study utilized a single group design with pretest, post-test and a three- and six-month follow-up, with measures of severity of GD and mood. The participants (*n* = 28) were treatment-seeking adults with GD, aged 17 to 49 years.

**Results:**

The results show a statistically significant decrease in symptoms of GD after treatment. Hours of gaming per week also decreased concomitantly with a 100% increase in non-gaming leisure hours. The decrease in symptoms of GD was maintained at the 3-months follow-up after treatment. Correspondingly we saw a decrease in both depression and anxiety that also was upheld 3 months after treatment.

**Conclusion:**

As GD is a new diagnostic concept more research is needed, also taking psychiatric comorbidity into consideration, to arrive at evidence-based conclusions regarding effective treatments. Considering the promising results in this small pilot study with large behavioral changes and reduced symptoms of GD, upheld at least 3 months after treatment, a larger randomized controlled study is warranted.

**Clinical Trial Registration:** https://www.clinicaltrials.gov/ct2/show/study/NCT05328596?term=NCT05328596&draw=2&rank=1, identifier NCT05328596.

## Introduction

In 2019, gaming disorder (GD) was included as a new diagnosis in the International Classification of Diseases (ICD-11) under the section for addiction (1). Gaming disorder is manifested by impaired control over gaming, increasing priority given to gaming and continuation or escalation of gaming despite the occurrence of negative consequences. In the fifth version of the Diagnostic and Statistical Manual of Mental Disorders (DSM-5), a similar construct named Internet Gaming Disorder was included among “Conditions for further studies” (2). Proposed criteria for this diagnosis include preoccupation, withdrawal, tolerance, reduced control, giving up other activities, continuing despite problems, deception, gaming to escape negative moods and risking or having lost relationships or opportunities. The suggested threshold for diagnosis is to fulfil at least five of these nine criteria in a 12-month period. As a consequence of being a newly defined disorder data on prevalence are scarce and inconsistent. Average worldwide prevalence of GD has been estimated at 1.96% with considerable differences between countries (3). Higher prevalence rates have been reported in specific groups, for example professional gamers with a prevalence rate of almost 4% (4).

There is evidence that GD often is accompanied by psychiatric comorbidity. A recent systematic review reported correlations between GD and anxiety, depression, ADHD, social phobia/anxiety, and obsessive–compulsive symptoms, with especially strong associations in adult populations (5). Regarding symptoms of ADHD, it is especially symptoms of inattention that are associated with GD (6). There are further findings that GD is associated with poor psychosocial functioning and lower performance in the academic or working spheres (7–9). Reduced self-satisfaction outside of playing video games, feelings of loneliness (10–12), negative affectivity and disinhibition (13) is also common. Whether these psychopathologies and impairments are risk factors for GD or consequences thereof, is not known and needs to be further studied longitudinally.

Gaming disorder is more common among men (3, 7, 14, 15) and among youth and young adults (3, 16). Low levels of family cohesion have been identified as a risk factor for GD in young adults and there is also a higher probability in this group of being unmarried, unemployed, having high levels of depression and anxiety (16–18), and a higher risk for suicide attempts (18) compared to individuals without GD. Several studies have shown that it is common among those with GD to use gaming to escape from negative emotions (18–20).

Cognitive behavioral therapy has been suggested as the most effective treatment for GD but has mostly been tested in a young population (21). There is also a scarcity of peer reviewed clinical treatment studies that include follow-up data to conclude if treatment gains are upheld over time (22). Regarding treatments for adults there are few studies that have evaluated the efficacy of a manualized CBT program (23–28) although both Wölfling et al. (25, 26) and Young (27, 28) designed their treatments for the broader concept of internet addiction (IA) including, i.e., online pornography and generalized internet addiction and not specifically for GD.

In an early CBT study that included 128 adults, decreased symptoms of IA was found and sustained at the six-month follow-up (27, 28). No control group was included. Further, the Short-term treatment of internet and computer game addiction (STICA) was tested in 143 young adults and improvements in symptoms related to IA was found compared to waitlist controls (26). In a non-randomized study, a CBT-approach for GD was compared with supportive therapy in 205 adults and reduced symptoms of GD was seen with results favoring CBT (23). Moreover, a multimodal treatment with CBT elements was tested in 40 adults and the severity of GD was decreased (24). Other CBT based psychotherapy studies has been conducted with younger subjects (12–22 years old), with 9 to 56 participants in each study, also showing positive results post treatment (29–34). Only three studies had a follow up period of three or six months (29, 30, 32).

Globally, more treatment research on GD is needed. It is therefore important to develop treatment manuals designed for this group of patients and evaluate their effects. This pilot study aimed to evaluate the effects and feasibility of a recently developed CBT treatment manual designed specifically for the treatment of GD. We first hypothesize that a 15-week CBT treatment will reduce symptoms of GD in a clinical population of young adults and adults fulfilling criteria for GD. We also hypothesize that a reduction of GD symptoms will be accompanied by a reduced amount of hours spent gaming each week. Our secondary hypothesis is that there will be a concurrent decrease in symptoms of psychiatric comorbidity such as depression and anxiety. We also have a third explorative hypothesis that the participants will experience an increased quality of life and have fewer symptoms of procrastination after treatment.

## Materials and methods

This is a single group pilot study with pretest, post-test and a three-month follow-up. The study included 28 participants and was conducted from February 2020 to March 2023 (from inclusion of first participant to the last three-month follow-up) in Gothenburg, Sweden, at the Clinic for Gambling Addiction and Screen Health (Mottagning för spelberoende och skärmhälsa), Department of Addiction and Dependency, Sahlgrenska University Hospital, Region Västra Götaland. The clinic is the largest of its kind in Sweden offering specialized care for patients with gambling and gaming disorder. Patients were referred to the clinic either via self-referral or by other healthcare facilities. The treatment lasted for 15 weeks. After 3 months the patients were followed up with questionnaires. A smaller amount of participants were also followed-up after 6 months.

### Subject recruitment and screening

The participants were consecutively recruited from the treatment-seeking population at the clinic. The initial assessment was conducted either as a videoconference or on site and included an anamnestic interview, a semi-structured diagnostic interview regarding symptoms of GD, screening for other psychiatric disorders, assessment of health, lifestyle, and psychosocial resources. After this assessment, made by a psychologist, a social worker or a nurse, participants were offered to enter the treatment program. All participants signed a consent form.

To be included in the study participants needed to fulfil the diagnostic criteria for Internet Gaming Disorder according to DSM-5 (≥ 5 criteria). Participants had to be able to read and write Swedish fluently and have turned 15 years old. Participants were excluded if they had somatic or psychiatric conditions that contraindicated treatment or severely hindered treatment participation e. g. ongoing psychotic, manic or hypomanic episode, severe depression (PHQ 20–27 p) or neurodevelopmental disorder (e.g., ADHD or autism) with low functional status evident by for example being in need of help with many activities of daily living, were currently in another psychological treatment with similar content as the one offered in the study or had started, or had ended or adjusted a medication for a psychiatric condition during the last 3 weeks. The study was approved by the regional ethics committee of the University of Gothenburg and complied with the guidelines of the Declaration of Helsinki (Dnr: 2020-07144).

### The CBT-treatment for gaming disorder

There exists no gold-standard treatment of behavioral addictions. The Gothenburg Treatment for Gaming Disorder (GOT-TO-GO) is designed to focus on gaming specific problems. The treatment has been developed at the clinic and consists of CBT-techniques such as stimulus control, cognitive restructuring and relapse prevention commonly known from other treatment programs for GD (24, 32), addictive behaviors (25, 26, 32, 35–38) and substance use disorders (39–41) including behavioral self-control training (42). In addition, elements from motivational interviewing (MI) (43) are used, especially in the initial stages of treatment to strengthen the motives for behavioral change, as this method has been shown to be effective in supporting other types of behavioral change (44, 45). Motivational interviewing does probably not have a significant effect as a standalone intervention (46). However, using MI-exercises such as “decisional balance” serves as a useful framework to chart both positive and negative aspects of gaming, thereby laying groundwork for the formulation of individualized treatment goals.

The manual tested in this study was delivered with one session per week comprising 15 weeks. Additional support regarding psychosocial resources or health and lifestyle factors were offered if such a need was identified. The added support consisted of a few optional sessions (described more in detail below), in addition to the CBT-treatment.

To closely follow the patients’ progression during treatment, they answered self-report questionnaires (dependent measures, see below) throughout the study (at baseline, mid-treatment, end of treatment and at follow-up). Starting at the first session, and continuing throughout the treatment, patients were also encouraged to keep track of their gaming activity via a weekly gaming diary.

The GOT-TO-GO treatment is divided in three phases: Initial stages, new skills and relapse prevention (for an overview see Table 1). *Phase 1*: In the initial phase individual goals for the treatment are formulated. Motivational techniques are also used to strengthen the patient’s commitment to change. Goals are formulated both regarding gaming activity (what amount and type of gaming activity the patient wants to retain at end of treatment) and other changes the patient wants to make during treatment (for example to increase weekly exercise or to increase social activities outside gaming). Self-monitoring of gaming is introduced. *Phase 2*: Sessions follow with a focus on learning new skills to control gaming activity and to initiate other activities. The patients learn to identify their individual triggers for gaming, and strategies for stimulus control are implemented (for example uninstalling programs, moving the computer to another room, or blocking internet access for parts of the day). Much attention is also devoted to the introduction of new activities (behavioral activation), chosen individually to match the interests and goals of each patient, to fill some of the time otherwise devoted to gaming. Techniques for handling difficult feelings and unhelpful thoughts related to gaming are also introduced and practiced, as well as time-management and problem-solving skills. *Phase 3*: At the final stages a plan is formulated to maintain the changes made during treatment and how to get back on track if a relapse occurs. A summary is made of the most helpful techniques learned during treatment, the patients identify situations where they expect it would be especially difficult to maintain their changes and formulate strategies to tackle this (both proactive to stay on track and reactive to get back on track if they relapse). *Follow up*: After treatment is completed, the patients are contacted by phone for a follow-up after three and six months. As part of the follow-up the patients also fill out self-report questionnaires. If needed, two booster sessions are offered to analyze problematic situations that have occurred and to revise the relapse prevention plan. *Optional modules*: In addition to the above-mentioned sessions, there are also optional modules. Based on the intake assessment an individual plan for optional modules is made. An individual patient can take part in none, some, or all of these. The optional modules consist of (a) 1–3 family sessions where family members and/or significant others meet with the patient and, with assistance from a social worker, make plans on how to work together to reach the patient’s treatment goals. This has been added as familial conflicts about gaming and lack of consensus about treatment goals might hinder change (47), and conversely that higher levels of family cohesion seem to be a protective factor against GD (16), (b) 1–3 additional sessions for support regarding psychosocial resources, for example to establish contact with other societal support systems, (c) 1–3 additional sessions for support regarding health and lifestyle factors, for example to initiate physical exercise or to cut down on alcohol use, (d) 1 additional session for support on how to plan and conduct home-work assignments throughout treatment.

**Table 1:** Content of the CBT-treatment for gaming disorder together with measure points.

Phase	Theme	Interventions	Measures
First visit	Assessment	Diagnostic assessment of Internet Gaming Disorder according to DSM-5 Anamnestic information Assessment of other psychiatric conditions Assessment of psychosocial resources Assessment of health and lifestyle factors	Baseline
Phase 1: Initial stages (Session 1–2)	Introduction and goal setting	Motivational interviewing Goal setting Introduction of self-monitoring strategies	Weekly gaming calendar
Phase 2: New skills (Session 3–12)	Learning new skills to gain control over the gaming activity and to initiate alternate activities	Psychoeducation Identification of individual triggers for gaming Time-management Stimulus control Behavioral activation Using skills from gaming to reach treatment goals Strategies to identify and handle feelings Strategies to identify and handle unhelpful thoughts Problem solving	Weekly gaming calendar + Mid-treatment
Phase 3: Relapse prevention (Session 13–15)	Making plans to maintain changes	Evaluation of treatment Individual plan to maintain changes and to handle relapses	Weekly gaming calendar + End of treatment
Follow-up		Follow-up on how changes have been maintained If needed: 2 booster sessions to revise the relapse prevention plan	Follow-up after 3 and 6 months
Optional	Additional support	Family sessions (1–3 sessions with the patient and his/her family members to formulate a plan on how to work together to reach the patient’s treatment goals). Additional session for support regarding psychosocial resources Additional session for support regarding health and lifestyle factors 1 session with support on how to work with home-work assignments	

The GOT-TO-GO manual is based on general techniques from other CBT-treatments for substance use disorders and behavioral addictions. Therefore, several parts are similar to other CBT-treatments [for example the method developed by Wölfling et al. (26)]. However, our manual also differs in many ways from other treatments for behavioral addictions and specifically gaming disorder. One essential difference is that the manual, unlike many other approaches, is specifically developed for gaming disorder. More specific differences are that strategies to control and limit gaming is implemented without a period of total abstinence, the manual consists of fewer sessions [15 sessions in total compared with 23 sessions described by Wölfling et al. (26)], and in the gaming diary, time spent gaming is separated from other types of time spent online. The intervention has been developed with a population with considerable psychiatric co-morbidity in mind. Handouts for patients have been made as simple as possible and a flexible system with additional sessions to meet individual needs has been designed. We also include family sessions to help the family support the patient and offer support to activate a professional network around the patient.

### Variables and measures

#### Primary outcome measures

##### Gaming addiction identification test

The GAIT was our main outcome measure. GAIT is the only screening tool for GD developed and validated in a Swedish population. It consists of 17 questions regarding gaming that cover all the DSM-5 diagnostic criteria for Internet gaming disorder. The questions concern all digital games including games on computer, mobiles or TV, both gaming with others and alone (48). Suggested cut-off for GD is at least five questions being endorsed as “completely agree.” For this study a version of the questionnaire has been used that covers gaming during the past 30 days. The 30-day version was used due to our repeated measure design with the aim to detect a change in symptoms of gaming disorder over the course of the 15-week treatment, as well as during follow-up at intervals of only 3 months. GAIT has very good internal consistency (Cronbach’s alpha = 0.95).

##### Gaming disorder – time line follow back

We used a timeline follow back measure as our second main outcome measure. This type of measure was originally developed to track alcohol-consumption (49) but has been adapted for this study to track behaviors relevant to GD. The GD-TLFB is a diary where frequency and duration of weekly gaming can be tracked as well as other time spent online and time spent on screen-free activities. The gaming diary serves as a valuable complement to the symptom measures. Although the aim of treatment is to alleviate the negative consequences of gaming (the symptoms) and not time spent gaming *per se*, still, decreasing time spent gaming is a necessary step to reach that goal. Aside from being used as an outcome measure, the gaming diary also serves as an important clinical tool for self-monitoring.

#### Secondary measures

The Patient Health Questionnaire (PHQ-9) consists of nine items screening for symptoms of depression during the last 2 weeks. PHQ-9 is developed according to the diagnostic criteria in DSM-4 and the total score can be used to assess severity of depressive symptoms. Based on the total score the level of severity is classified as none (0–4), mild (5–9), moderate (10–14), moderately severe (15–19) or severe (20–27) depression. PHQ-9 has been shown to have high validity in detecting severity of depression (50).

The Generalized Anxiety Disorder Assessment (GAD-7) was developed as an instrument to measure severity of symptoms of anxiety. It is a seven-item questionnaire screening for symptoms during the last 2 weeks. The total score is 21, and the scores indicate minimal (0–4), mild (5–9), moderate (10–14) or severe (15–21) anxiety (51).

#### Exploratory measures

The Brunnsviken Brief Quality of life scale (BBQ) measures an individual’s subjective quality of life. It is divided into six different life areas that are rated individually regarding perceived importance and satisfaction. The maximum score is 96 with higher scores indicating higher levels of quality of life, and scores below 52 being associated with clinical samples (52).

The Pure Procrastination Scale (PPS) identifies the occurrence and severity of procrastination. It consists of 12 items rated on a 1–5 point Likert scale with higher scores indicating higher levels of procrastination (53).

#### Subject demographics

We also collected demographic data about the participants including age, sex, educational level, living situation and current occupation. Levels of alcohol and drug use were measured with the AUDIT (54) and DUDIT (55).

The Alcohol Use Disorders Identification Test (AUDIT) is a screening tool for alcohol related problems and identifies individuals with harmful use of alcohol. It consists of 10 items divided into three areas: alcohol consumption, symptoms of dependence and negative consequences of alcohol consumption. The maximum score is 40, with a cut-off score of 6 for women and 8 for men indicating hazardous or harmful drinking (54).

Drug Use Disorders Identification Test (DUDIT) is a screening tool for problematic use of illicit drugs. Like AUDIT, it is a 10-item instrument with a maximum score of 40. The questions are categorized in three areas, drug use, dependence symptoms and negative consequences of drug use. DUDIT scores of 1 or more for women and 3 or more for men indicate problematic drug use (55).

#### Data analysis

All analyses were conducted in IMB SPSS 28.0.1.1. Out of the 28 participants 12 were treated individually and the rest in group format. In the analyses, irrespective of having received the treatment via group or individual sessions, data from all participants have been included. The primary outcome variables were symptoms of GD measured by the GAIT and the four measures included in the gaming diary. Secondary measures included the PHQ-9, the GAD-7, and the exploratory measures were the BBQ and the PPS. The gaming diary consisted of nine repeated measures from the start of treatment to the last session of treatment. The GAIT, as well as the PHQ-9 and GAD-7 were also given at repeated intervals, at baseline, mid-treatment, termination of treatment, and 3-months follow-up. The BBQ and PPS instead were repeated at baseline, termination, and 3-months follow-up. For the gaming diary a total of 41% of data points were missing, ranging between 7% to at most 50% at specific timepoints and measures. For the GAIT, PHQ-9, and GAD-7 a total of 33% of data were missing, ranging from 4% at baseline to 57% at the 3-month follow-up. For the BBQ a total of 26% of data was missing, ranging from 4% at baseline and 57% at three-month follow-up. For the PPS a total of 42% of data was missing, ranging from 29% at baseline and 61% at 3-month follow-up.

Mixed-effects models fitted with maximum likelihood estimation were used to estimate individual changes over time during treatment (gaming diary) and from baseline to follow-up (GAIT, PHQ-9, GAD-7, BBQ, and PPS). Mixed effects models were used as they handle missing data and correlation between repeated measurements better than a classical repeated measures ANOVA (56, 57). Further, as the mixed model uses all available data points it is possible to do an intention-to-treat analysis, including all participants in the analysis.

For the primary outcome measure GAIT, a basic model with a fixed slope for *time* was created. *Time* was coded as 0–3 with 0 being baseline and 3 being 3-month follow-up. To account for possible non-linear effects a quadratic effect of *time x time* was tested, found non-significant and was therefore discarded. A random intercept and random slope for *time* was tested but did not improve the model according to a likelihood ratio test and were thus discarded. A heterogeneous first-order autoregressive covariance pattern was used for the repeated measures and was significant, *p* < 0.001.

Model building was approached in the same way for the secondary outcome PHQ-9, and *time* was similarly coded here. The quadratic effect of *time x time* was non-significant and discarded. A random intercept and random slope for *time* improved model fit according to a likelihood ratio test *p* < 0.05 and were retained. A diagonal covariance pattern was used for the repeated measures as the model did not otherwise converge. Unstructured covariance type was used for the random effects.

For the GAD-7 *time* was similarly coded. The quadratic term of *time x time* was non-significant and discarded. The random intercept and random slope for *time* did not improve model fit according to a likelihood ratio test and were discarded. A heterogeneous first-order autoregressive covariance pattern was used for the repeated measures and was significant, *p* < 0.001.

For the BBQ *time* was coded 0–2, with 0 being baseline, and 2 being three-month follow-up. The quadratic term of *time x time* was non-significant and discarded. When the random intercept and random slope for *time* was added the model failed to converge, and the model with random intercept were not an improvement according to a likelihood ratio test. The random effects were thus discarded. A heterogeneous first-order autoregressive covariance pattern was used for the repeated measures and was significant, *p* < 0.001.

For the PPS *time* was similarly coded. The quadratic term of *time x time* was non-significant and discarded. When the random intercept and random slope for time was added the model failed to converge. A model with diagonal covariance pattern and random intercept did converge and was a better model according to a likelihood ratio test. However, a model without random effects using a heterogeneous first-order autoregressive covariance pattern (*p* < 0.001) proved to have similar fit and was chosen as it was a simpler model.

As the 6 months follow-up was not conducted for all participants (*n* = 9 out of 28, *n* = 8 for the GAD-7) this timepoint was not included in the above models. The means and standard deviations for these participants are however presented for descriptive purposes.

For the primary outcome hours gaming/week a basic model with a fixed slope for *time* was created. *Time* was coded as 0–8 with 0 being diary entry pre-treatment and 8 the final entry during treatment. A quadratic fixed effect of *time x time* was tested to account for possible non-linearity. This effect was significant, *p* < 0.05 and was retained. A random intercept improved model fit according to a likelihood ratio test, *p* < 0.001 and was retained. A heterogeneous first-order autoregressive covariance pattern was used for the repeated effects and was significant, *p* < 0.001.

For days gaming/week model building was approached in the same way and *time* was coded similarly. The quadratic effect of *time x time* was non-significant and was discarded. A random intercept and random slope for *time* significantly improved model fit (likelihood ratio test, *p* < 0.001) and were retained. A diagonal covariance pattern was used for the repeated measures as the model did not otherwise converge. Unstructured covariance type was used for the random effects.

For non-gaming screen time, *time* was coded similarly. The quadratic effect was non-significant and discarded. A random intercept and random slope for *time* produced a model with better fit (likelihood ratio test, *p* < 0.001) and these effects were retained. A diagonal covariance pattern was used for the repeated measures as the model did not otherwise converge. Unstructured covariance type was used for the random effects.

For non-screen leisure time, *time* was coded in the same way. The quadratic effect was non-significant and discarded. A random intercept and random slope for *time* significantly improved model fit (likelihood ratio test, *p* < 0.001) and these effects were retained. A heterogeneous first-order autoregressive covariance pattern was used for the repeated effects and was significant, *p* < 0.05. Unstructured covariance type was used for the random effects.

Estimated means of variables were calculated for all time points in the mixed-models that yielded significant effects. Estimated means of non-significant models are not reported.

Cohen’s d effect sizes were calculated for significant effects (58). For the mixed-models, Cohen’s d was calculated between baseline mean and mean at the final time-point in the series using the model estimated means together with the observed standard deviation at baseline (59). Confidence intervals of within group effect sizes were calculated using Pearson correlations of observed values between baseline and the final time-point.

In the participants section, harmful alcohol use and problematic drug use was based on cut-off values for AUDIT (≥ 8 for men, ≥ 6 for women) and DUDIT (≥ 3 for men, ≥ 1 for women) scores (54, 55).

## Results

### Subject demographics

There were 28 participants included in this study, with an average age of 27.7 (SD 7.3) years. Of these, there was only one woman (3.6%), and the rest were men. The most preferred games were Massively multiplayer online role-playing games (MMORPG), Multiplayer online battle arena (MOBA) and First-person shooter games (FPS). In the sample, 71.5% had a high school education or higher. The majority 60.8% were employed or studying, 17.9% were on sick leave, 14.3% were unemployed and 7.2% had another occupation or some combination of the above. The most common living situation was living together with relatives/parents/friends (44.4%), followed by living alone (22.2%). Of the participants, 26.9% used nicotine in some form. Regarding alcohol and illicit drugs, 11.1% of participants had a harmful alcohol use based on AUDIT scores, and 11.1% a problematic drug use based on DUDIT scores. See Table 2 for a full list of subject demographics.

**Table 2:** Subject demographics.

Demographic variables	Total sample (*n* = 28)^a^
Age M (SD)	27.7 (7.3)
Age range	17–49
Gender %	
Men	96.4
Women	3.6
Preferred game genres (n)^b^	
MMORPG	10
MOBA	7
FPS	10
Other	9
Education %	
Less than high school	28.6
High school	28.6
Occupational training	17.9
University	25.0
Occupational status %	
Working	42.9
Sick-leave	17.9
Unemployed	14.3
Studying	17.9
Other/combination of above	7.2
Living Situation %	
Alone	22.2
With partner	18.5
With relatives/friends	44.4
Single parent	3.7
With partner and children	11.1
Nicotine use %	
Yes	26.9
No	73.1
Harmful alcohol use %^c^	
Yes	11.1
No	88.9
Problematic drug use %^d^	
Yes	11.1
No	88.9
Psychiatric co-morbidities^e^	
F10-F19 Substance use disorders	6
F20-F29 Schizophrenia etc.	1
F30-39 Mood disorders	21
F40-48 Neurotic disorders	8
F50-F59 Eating disorders etc.	3
F60-F69 Personality disorders	2
F80-F89 Autism etc.	2
F90-F98 ADHD etc.	7

^a^Partial missing data for living situation (*n* = 1), tobacco use (*n* = 2), AUDIT (*n* = 1) and DUDIT (*n* = 10). ^b^One individual can have several preferred game genres. ^c^Based on AUDIT scores. ^d^Based on DUDIT scores. ^e^Diagnostic categories according to classification with ICD-10. One individual can have several diagnoses.

Of the 28 participants, 24 completed the 15 week GOT-TO-GO treatment resulting in a dropout rate at 14% which is below the normal rates (19–51%) in psychiatric health care (60).

### Primary outcomes

**Figure 1:**
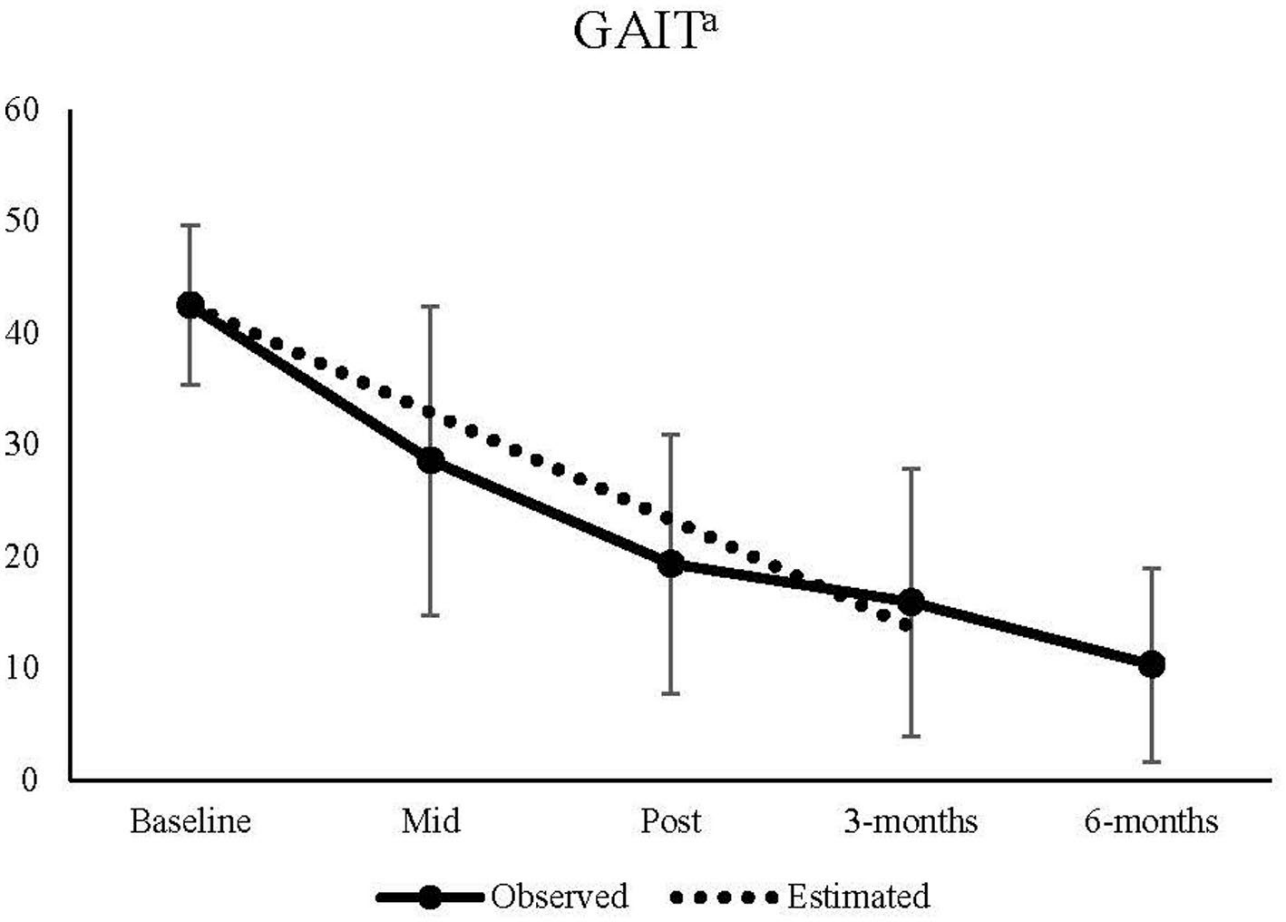
Observed means from baseline to 6-month follow-up, and estimated means from baseline to 3-months follow-up for the GAIT. ^a^A significant effect of time, *p* < 0.001 was found in the model.

The model estimates for the primary outcome of GD symptoms measured by the GAIT can be found in Table 3 along with confidence intervals, *p*-value and effect size. The model intercept of 42.52 is the estimated baseline score for all participants. The significant effect of time (*p* < 0.001) of −9.62 means that from each step between baseline to 3-month follow-up the GAIT score is reduced by X*9.62 points (baseline X = 0, mid X = 1, post X = 2, three-months X = 3). This means that symptoms of GD decreased over time. This is illustrated in Figure 1, where model estimated means are plotted over time and compared to observed means with standard deviations. The observed mean for the limited number of 6-months follow-ups is also presented in Figure 1 for descriptive purposes. The effect size of change between baseline to 3-month follow-up was large, *d* = 4.03.

**Table 3:** Model estimates of gaming behaviors, including confidence intervals, *p*-values and effect sizes.

Model	Estimate	95% CI	*p*-value
**GAIT**			
Intercept	42.52	39.64 to 45.39	< 0.001
Time (baseline to 3 months)	−9.62	−11.6 to −7.63	< 0.001
Within group effect size (Cohen’s d)	4.03^a^	−0.97 to 9.4	
**Hours/week**			
Intercept	45.65	33.99 to 57.32	< 0.001
Time (pre-treatment to treatment final entry)	−8.33	−12.27 to −4.39	< 0.001
Time x Time	0.65	0.03 to 1.0	< 0.001
Within group effect size (Cohen’s d)	0.63^b^	−0.99 to 2.08	
**Days/week**			
Intercept	5.57	4.65 to 6.49	< 0.001
Time (pre-treatment to treatment final entry)	−0.11	−0.28 to 0.05	= 0.164
Within group effect size (Cohen’s d)	N/A		
**Non-gaming screen hours/week**			
Intercept	25.87	20.58 to 31.16	< 0.001
Time (pre-treatment to treatment final entry)	−0.73	−1.33 to −0.14	< 0.05
Within group effect size (Cohen’s d)	0.36^b^	−1.91 to 2.49	
**Non-gaming leisure hours/week**			
Intercept	16.60	10.09 to 23.1	< 0.001
Time (pre-treatment to treatment final entry)	1.41	0.63 to 2.19	= 0.001
Within group effect size (Cohen’s d)	0.75^b^	−0.18 to 1.97	

^a^Effect size is calculated between baseline and 3-month follow-up for GAIT. ^b^Effect size is calculated between baseline and the final week of treatment for the gaming diary.

In Table 3 the model estimates together with *p*-values, confidence intervals and effect sizes for the various measures of gaming behavior derived from the gaming diary are reported. Participants were gaming at a model estimated average of 45.65 h/week at baseline. A significant effect of time (pre-treatment to final measurement) (*p* < 0.001) of −9.62 and time x time (*p* < 0.001) of 0.65 meant that hours/week were reduced during treatment, but the rate of change slowed down each week and even increased somewhat at the end of treatment (each step from baseline the score is reduced by X*8.33 – X^2^*0.65). The model estimated hours/week at the final measurement was an average of 20.61 h/week. The effect size of the reduction from pre-treatment to the final measurement was medium sized, d = 0.63. There was also a significant effect of time (*p* < 0.05) of −0.73 regarding non-gaming screen hours/week. This means they were reduced linearly from a model estimated average of 25.87 h pre-treatment to 20.0 h at the final measurement. The effect size was small, d = 0.36. Non-gaming leisure hours instead significantly increased linearly over time (*p* < 0.001) with 1.41 for each measurement point during treatment. The model estimated an average of 16.6 h non-gaming leisure time at baseline, and 27.89 h at the final measurement. This was a medium sized effect, d = 0.75.

No significant change over time (*p* = 0.164) was found regarding the number of days/week participants were gaming. See Table 4 for observed means and standard deviations, and model estimated means for the measures in the gaming diary.

**Table 4:** Time line follow back gaming diary.

Measure	Pre	Entry 1	Entry 2	Entry 3	Entry 4	Entry 5	Entry 6	Entry 7	Entry 8
**Hours/week**^b^									
Observed M^a^	51.71	40.08	29.62	24.79	22.82	16.9	18.81	16.93	19.5
Observed SD^a^	39.64	26.24	22.47	22.12	18.25	13.39	14.94	16.08	15.64
Estimated M	45.65	37.97	31.59	26.51	22.73	20.25	19.07	19.19	20.61
**Days/week**									
Observed M^a^	5.71	6.08	5.46	5.12	4.59	4.84	4.56	4.4	4.86
Observed SD^a^	2.34	2.02	2.35	2.33	2.56	2.43	2.39	2.67	2.66
Estimated M	N/A	N/A	N/A	N/A	N/A	N/A	N/A	N/A	N/A
**Non-gaming screen hours/week**^c^									
Observed M^a^	26.43	23.04	24.6	26.44	23.48	26.47	19.63	20.73	17.96
Observed SD^a^	16.37	14.63	11.73	11.15	13.2	16.93	11.21	8.87	7.95
Estimated M	25.87	25.13	24.4	23.67	22.93	22.2	21.46	20.73	20.0
**Screen-free lesiure time**									
Observed M^a^	12.89	17.81	21.81	26.91	26.45	31.89	30.63	31.7	34.36
Observed SD^a^	13.26	13.64	18.19	21.99	17.71	24.58	24.93	27.13	24.84
Estimated M	16.6	18.01	19.42	20.83	22.25	23.66	25.07	26.48	27.89

Means, standard deviations and model estimated means for all timepoints in the gaming diary. ^a^Based on all non-missing data. Missing data ranges from 7–50% at specific timepoints. ^b^A Significant effects of time, *p* < 0.001 and time x time, *p* < 0.05 were found in the model. ^c^A significant effect of time, *p* < 0.05 was found in the model. ^d^A significant effect of time, *p* < 0.001 was found in the model.

### Secondary and exploratory outcomes

Symptoms of depression were found to significantly (*p* = 0.001) decrease linearly over time, from baseline to 3-month follow-up with a rate of −2.44 points on the PHQ-9 for each timepoint (baseline, mid-treatment, post-treatment, 3-months). The model estimated mean at baseline was 10.64 and this was reduced to a model estimated mean at 3-month follow-up of 3.33. The effect size of change was large, d = 0.98. Anxiety symptoms measured by the GAD-7 also decreased linearly over time (*p* < 0.001) with an estimated rate of −1.42 from an estimated baseline score of 7.21 to 3.09 at 3-month follow-up. This was a large effect, d = 0.80.

Procrastination, measured by the PPS, also decreased significantly over time (*p* < 0.001) by −5.58 for each timepoint (baseline, post-treatment, 3-months) from a model estimated 42.25 at baseline to 31.1 at 3-month follow-up, which was a large effect, d = 0.99. There was no significant effect of time on quality of life measured by the BBQ from baseline to 3-month follow-up, *p* = 0.060.

Model estimates, *p*-values, confidence intervals and effect sizes for the PHQ-9, GAD-7, BBQ, and PPS models can be found in Table 5. Observed means and standard deviations as well as model estimated means can be found in Table 6. Observed means for the limited number of 6-month follow-ups are for descriptive purposes also presented in Table 6.

**Table 5:** Model estimates of non-gaming behaviors secondary outcomes, including confidence intervals, *p*-values and effect sizes.

Model	Estimate	95% CI	*p*-value
**PHQ-9**			
Intercept	10.64	8.0 to 13.29	< 0.001
Time (baseline to 3 months)	−2.44	−3.79 to −1.09	= 0.001
Within group effect size (Cohen’s d)^a^	0.98	−1.23 to 2.97	
**GAD-7**			
Intercept	7.21	5.33 to 9.1	< 0.001
Time (baseline to 3 months)	−1.42	−2.2 to −0.63	< 0.001
Within group effect size (Cohen’s d)^a^	0.80	−1.60 to 2.96	
**BBQ**			
Intercept	42.38	34.08 to 50.67	< 0.001
Time (baseline to 3 months)	5.33	−0.23 to 10.89	= 0.060
Within group effect size (Cohen’s d)^a^	N/A		
**PPS**			
Intercept	42.25	37.02 to 47.49	< 0.001
Time (baseline to 3 months)	−5.58	−8.35 to −2.8	< 0.001
Within group effect size (Cohen’s d)^a^	0.99	−0.49 to 2.44	

^a^Effect sizes are calculated between baseline and 3-month follow-up.

**Table 6:** Means, standard deviations, and model estimated means for non-gaming behavior secondary outcomes.

Measure	Baseline	Mid	Post	3 month	6 month^d^
**PHQ-9**^b^					
Observed M (SD)^a^	12.0 (7.44)	7.09 (5.45)	5.26 (4.57)	6.64 (8.03)	3.78 (3.38)
Estimated M	10.64	8.21	5.77	3.33	-
**GAD-7**^c^					
Observed M (SD)^a^	7.22 (5.31)	6.61 (4.6)	3.61 (3.37)	3.09 (2.74)	3.75 (5.9)
Estimated M	7.21	5.8	4.38	2.96	-
**BBQ**					
Observed M (SD)^a^	42.37 (21.37)	-	48.74 (18.7)	56.27 (16.73)	58.33 (23.89)
Estimated M	42.38	-	47.7	53.03	-
**PPS**^c^					
Observed M (SD)^a^	43.0 (11.24)	-	35.39 (12.76)	31.27 (15.4)	33.11 (18.2)
Estimated M	42.25	-	36.68	31.1	-

Observed and estimated means for the PHQ-9, GAD-7, BBQ and PPS from baseline to 3-month follow-up. The data are presented over time as means with standard deviations. Baseline = before treatment start, Mid = middle of treatment, Post = after treatment, 3-months = post treatment end. ^a^Based on all non-missing data. Missing data ranges from 4–61% at specific timepoints. ^b^A significant effect of time, *p* = 0.001 was found in the model. ^c^A significant effect of time, *p* < 0.001 was found in the model. ^d^Not included in the model. Means and standard deviations for *n* = 9 participants included for descriptive purposes.

## Discussion

This was an uncontrolled pilot study intended to evaluate the feasibility of a newly developed manualized CBT treatment for patients diagnosed with GD. The 28 participants included in the study were followed from baseline to 3-months post treatment. We investigated symptoms of GD, sociodemographic factors, alcohol and drug use, depression and anxiety, quality of life and procrastination.

### Sociodemographic characteristics

We notice both differences and similarities regarding sociodemographic characteristics when comparing the patients in our study with populations in earlier studies. The mean age in our sample was 28, meaning that we reached an older group than most previous clinical studies where the age range has been between 12 to 22 years of age (5, 29–34). However, both the age range and the high education level seen in our study is similar to other IA-studies with adult patients (26, 28).

In our study, only one woman chose to participate. The prevalence of GD is estimated to be 2.5 times higher among men than women, and therefore it is expected that more men than women will seek treatment. However, the proportion of women in our study and other treatment studies for GD (23, 24) are still much lower than could be expected based on prevalence. In this aspect, GD differs from other psychiatric conditions where women usually are overrepresented as treatment-seekers (61). Still, we believe it is important to continue including women in future treatment studies, and also make active efforts to reach more women with GD.

The association between GD and substance use has been investigated, but findings so far are mixed. We found that a small proportion of our patients had a problematic intake of alcohol or other drugs, to a comparable extent with the Wölfling et al. (26) study. Other studies have for example shown a positive correlation between severity of GD and frequency of substance use (63–65). Studies have also shown that those who play under the influence of for example stimulants, Ecstasy/MDMA, sedatives or amphetamines spend more time gaming than non-substance users (62) and that high alcohol consumption is an antecedent to gaming disorder (66). On the other hand, it has also been reported that a heavy investment in gaming may lead to a reduction in alcohol use (67) or that no association between alcohol and gaming disorder could be detected (63). Considering the findings that some treatment seekers with GD also have a problematic intake of alcohol or other drugs, together with the mixed research findings so far regarding associations between GD and substance use, we believe that it is important to regularly screen for possible co-morbidities with SUD in future treatment studies. Thereby, we can increase our knowledge on how substance use and SUD might affect treatment results, and if changes regarding gaming also are associated with changes in substance use.

### Changes during treatment

We found a significant reduction in symptoms of GD between baseline measurements and the 3-months follow-up, in total a decrease by 70% based on measures with the GAIT. Similarly, hours spent gaming per week, measured with the GD-TLFB, decreased by 62% during treatment, which corresponds to 32 h less gaming per week. Time spent gaming after treatment was on average 19.5 h per week which is well within the normal range according to Swedish Media Council (68). We want to emphasize that the aim of the treatment was not total abstinence from gaming or other internet activities but simply to gain control over gaming habits. With the gaming diary we also wanted to measure changes in non-gaming screen time, to make sure that time spent gaming not only transitioned into other types of screen-time. Instead, the gaming diary showed that the decrease in time spent gaming also was accompanied by a small decrease in other types of screen time. The patients also more than doubled their amount of screen-free leisure time. It is difficult to compare results from different studies as there are no gold-standard instruments for measuring GD, and many different instruments have been used in previous studies (69). With this caveat in mind, we observe that in our study, as well as in earlier studies regarding adults with IA (26, 28) and GD (23, 24), we see substantial changes in symptoms after treatment compared to baseline. This also holds for changes in hours spent online in our as well as in other studies (26, 28). In summary, this shows promise for using a CBT approach for treating GD.

Our secondary measures focused on anxiety, depression, quality of life, and procrastination. For these variables we saw changes in the expected direction, although the change in quality of life did not reach statistical significance. We argue that all these aspects are important to take into account when evaluating treatments for GD. By measuring for example quality of life we address a broader definition of health than simply the absence of symptoms, and capture additional aspects highly relevant to GD. Lower quality of life has been shown to be associated to GD, and also differentiating highly engaged gamers from those with problematic gaming (70, 71). The complex interplay between these factors is also illustrated by findings that levels of anxiety and depression mediate the relationship between GD and quality of life (72).

We also saw a significant reduction of symptoms of procrastination, measured by the PPS (53) after treatment, although the levels were still high. The decision to include strategies to identify and handle procrastination in our manual was based both on clinical observations, and earlier findings that symptoms of procrastination was associated with clinical severity of internet gaming disorder (73). Similarly, in a prevention program for adolescents with at risk for GD, a reduction of symptoms of GD was accompanied by a decrease in procrastination (74). The association between procrastination and GD is further supported by findings that lower levels of procrastination predict spontaneous remission of GD (75). Based on this we suggest that procrastination could be a relevant factor to take into consideration in treatment strategies for GD.

### Limitations, implications, future research, and conclusions

Our study had some clear limitations but also strengths. There are a number of limitations in the dataset from this study: the sample size is small, there are missing data, there is no pre-treatment measurement for the primary outcome and a number of secondary outcomes, and repeated measurements have been given at variable time points (i.e., the gaming diary was not given every week during treatment but instead at specific sessions with varying amounts of time in between). The choice to collect the gaming diary more seldom than every week was made to minimize missing data. Still, a substantial amount of data was missing. In the coming randomized controlled trial (RCT) we will amend this by focusing more on collecting diaries on even fewer occasions during treatment, thereby being able to focus more on making sure that diaries on these chosen weeks will be registered. The use of weekly diaries will still be part of the treatment, but our experiences so far indicate that, for a considerable part of the intended study population, remembering or wanting to complete these daily or weekly throughout the whole treatment period poses a challenge. Even though statistical methods (maximum likelihood estimation) have been employed to reduce the problem of missing data, the results of this pilot study should be interpreted with care. The single group design also limits the conclusions. These limitations will be corrected in a randomized controlled treatment study with follow-up at 3, 6, 12, 18 and 24 months (ClinicalTrials.gov NCT05328596).

There is a lack of treatment options and insufficient evidence regarding effective treatment of GD. This is the first treatment manual for GD, developed and studied in Sweden, closely evaluated with standardized measures and one of the few treatments so far developed specifically for GD. Moreover, our study participants have undergone a careful diagnostic assessment. This study is also highly clinically relevant as the participants are treatment seeking patients in regular care. Moreover, the patients have completed a follow up assessment 3 months after the treatment ended, which gives us a longitudinal indication of sustained effects. This is a strength since follow-up data after treatment is scarce (3, 21). Findings about the stability of GD over time are somewhat mixed. From studies to date it seems that a proportion of people with GD spontaneously recover (76) but a sizable amount remains that still fulfil the diagnosis at least one year later or more (66, 75). We also consider it a strength that we offer a flexible treatment, with additional sessions to add if needed.

To regain control over one’s gaming behavior is challenging for all individuals and even harder for those with comorbid psychiatric disorders. We noted that almost 100% of the participants in our study had symptoms of psychiatric comorbidity with mood disorders as the most common one. A vast majority were men, not seldom isolated using the game to escape from negative thoughts and emotions.

Our CBT treatment, specifically designed to treat patients with GD, showed promising results with reduced symptoms of GD, upheld at least 3-months after treatment, accompanied by decreased time spent gaming almost equivalent to a normal work week. We further observed that the treatment was feasible to deliver as most patients stayed in treatment, and that the treatment was possible to implement as a part of regular care at the treatment center.

In conclusion, there is insufficient evidence regarding effective treatments for GD. Based on our promising preliminary pilot findings, we will conduct a RCT. For the upcoming RCT the manual will be shortened, giving increased possibilities to add sessions based on individual needs. We believe there is a need for a flexible treatment specifically designed for individuals with GD with considerable psychiatric comorbidity, to help them improve their quality of life and regain control over their gaming.

## Data availability statement

The raw data supporting the conclusions of this article will be made available by the authors, without undue reservation.

## Ethics statement

The studies involving human participants were reviewed and approved by Swedish Ethical Review Authority. Written informed consent from the participants' legal guardian/next of kin was not required to participate in this study in accordance with the national legislation and the institutional requirements.

## Author contributions

AH had the main responsibility of the writing of the manuscript also contributed with specific knowledge in gaming addicted patients and the main idea for the manuscript. MM made all the statistical analyses and responsible for the result section. EA was responsible for the informed consent form to collect and keep track of the data and Method section. SL, JM, and AL developed the GOT-TO-GO manual and worked as the psychologists treating the patients in the study, reading the manuscript, and helped in writing the Method section. AS was a senior researcher of the work and the Principal investigator for this research, and supervised the writing of the whole manuscript throughout the research process. All authors contributed to the article and approved the submitted version.

## Funding

This study was supported by Svenska Spel’s Independent Research Council, grant no FO2021-0007.

## Conflict of interest

The authors declare that the research was conducted in the absence of any commercial or financial relationships that could be construed as a potential conflict of interest.

## Publisher’s note

All claims expressed in this article are solely those of the authors and do not necessarily represent those of their affiliated organizations, or those of the publisher, the editors and the reviewers. Any product that may be evaluated in this article, or claim that may be made by its manufacturer, is not guaranteed or endorsed by the publisher.

## References

[1] WHO. (2019). *International Classification of Diseases 11th revision* [online]. Internet. Available: www.icd.who.int/en (Accessed October 18, 2022)

[2] American Psychiatric Association. Diagnostic and statistical manual of mental disorders DSM-5. Arlington, VA: American Psychiatric Association (2013).

[3] Stevens MW Dorstyn D Delfabbro PH King DL. Global prevalence of gaming disorder: a systematic review and meta-analysis. Aust N Z J Psychiatry. (2021) 55:553–68. doi: 10.1177/0004867420962851, PMID: 33028074

[4] Maldonado-Murciano L Guilera G Montag C Pontes HM. Disordered gaming in esports: comparing professional and non-professional gamers. Addict Behav. (2022) 132:107342. doi: 10.1016/j.addbeh.2022.107342, PMID: 35584554

[5] González-Bueso V Santamaría JJ Fernández D Merino L Montero E Ribas J. Association between internet gaming disorder or pathological video-game use and comorbid psychopathology: a comprehensive review. Int J Environ Res Public Health. (2018) 15:668. doi: 10.3390/ijerph15040668, PMID: 29614059 PMC5923710

[6] Dullur P Krishnan V Diaz AM. A systematic review on the intersection of attention-deficit hyperactivity disorder and gaming disorder. J Psychiatr Res. (2021) 133:212–22. doi: 10.1016/j.jpsychires.2020.12.026, PMID: 33360866

[7] Beutel ME Hoch C Wölfling K Müller KW. Clinical characteristics of computer game and internet addiction in persons seeking treatment in an outpatient clinic for computer game addiction. Z Psychosom Med Psychother. (2011) 57:77–90. doi: 10.13109/zptm.2011.57.1.77, PMID: 21432840

[8] Brunborg GS Mentzoni RA Frøyland LR. Is video gaming, or video game addiction, associated with depression, academic achievement, heavy episodic drinking, or conduct problems? J Behav Addict. (2014) 3:27–32. doi: 10.1556/JBA.3.2014.002, PMID: 25215212 PMC4117274

[9] Haghbin M Shaterian F Hosseinzadeh D Griffiths MD. A brief report on the relationship between self-control, video game addiction and academic achievement in normal and ADHD students. J Behav Addict. (2013) 2:239–43. doi: 10.1556/JBA.2.2013.4.7, PMID: 25215206 PMC4154570

[10] Bargeron AH Hormes JM. Psychosocial correlates of internet gaming disorder: psychopathology, life satisfaction, and impulsivity. Comput Hum Behav. (2017) 68:388–94. doi: 10.1016/j.chb.2016.11.029

[11] Bender PK Gentile DA. Internet gaming disorder: relations between needs satisfaction in-game and in life in general. Psychol Popular Media. (2020) 9:266–78. doi: 10.1037/ppm0000227

[12] Yau YH Potenza MN. Gambling disorder and other behavioral addictions: recognition and treatment. Harv Rev Psychiatry. (2015) 23:134–46. doi: 10.1097/HRP.0000000000000051, PMID: 25747926 PMC4458066

[13] Müller KW Werthmann J Beutel ME Wölfling K Egloff B. Maladaptive personality traits and their interaction with outcome expectancies in gaming disorder and internet-related disorders. Int J Environ Res Public Health. (2021) 18:3967. doi: 10.3390/ijerph18083967, PMID: 33918737 PMC8070224

[14] Chen KH Oliffe JL Kelly MT. Internet gaming disorder: an emergent health issue for men. Am J Mens Health. (2018) 12:1151–9. doi: 10.1177/1557988318766950, PMID: 29606034 PMC6131461

[15] Darvesh N Radhakrishnan A Lachance CC Nincic V Sharpe JP Ghassemi M. Exploring the prevalence of gaming disorder and internet gaming disorder: a rapid scoping review. Syst Rev. (2020) 9:68. doi: 10.1186/s13643-020-01329-2, PMID: 32241295 PMC7119162

[16] Adams BLM Stavropoulos V Burleigh TL Liew LWL Beard CL Griffiths MD. Internet gaming disorder behaviors in emergent adulthood: a pilot study examining the interplay between anxiety and family cohesion. Int J Ment Heal Addict. (2018) 17:828–44. doi: 10.1007/s11469-018-9873-0

[17] Burleigh TL Stavropoulos V Liew LWL Adams BLM Griffiths MD. Depression, internet gaming disorder, and the moderating effect of the gamer-avatar relationship: an exploratory longitudinal study. Int J Ment Heal Addict. (2017) 16:102–24. doi: 10.1007/s11469-017-9806-3

[18] Kim DJ Kim K Lee H-W Hong J-P Cho MJ Fava M. Internet game addiction, depression, and escape from negative emotions in adulthood: a Nationwide Community sample of Korea. J Nerv Ment Dis. (2017) 205:568–73. doi: 10.1097/NMD.0000000000000698, PMID: 28598958

[19] Bäcklund C Elbe P Gavelin HM Sörman DE Ljungberg JK. Gaming motivations and gaming disorder symptoms: a systematic review and meta-analysis. J Behav Addict. (2022) 11:667–88. doi: 10.1556/2006.2022.00053, PMID: 36094861 PMC9872536

[20] Király O Billieux J King DL Urbán R Koncz P Polgár E. A comprehensive model to understand and assess the motivational background of video game use: the gaming motivation inventory (GMI). J Behav Addict. (2022) 11:796–819. doi: 10.1556/2006.2022.00048, PMID: 35939353 PMC9872527

[21] King DL Delfabbro PH Wu AMS Doh YY Kuss DJ Pallesen S. Treatment of internet gaming disorder: an international systematic review and CONSORT evaluation. Clin Psychol Rev. (2017) 54:123–33. doi: 10.1016/j.cpr.2017.04.002, PMID: 28458097

[22] Zajac K Ginley MK Chang R. Treatments of internet gaming disorder: a systematic review of the evidence. Expert Rev Neurother. (2020) 20:85–93. doi: 10.1080/14737175.2020.1671824, PMID: 31544539 PMC6930980

[23] Han J Seo Y Hwang H Kim SM Han DH. Efficacy of cognitive behavioural therapy for internet gaming disorder. Clin Psychol Psychother. (2020) 27:203–13. doi: 10.1002/cpp.2419 31881100

[24] Sharma MK Anand N Tadpatrikar A Marimuthu P Narayanan G. Effectiveness of multimodal psychotherapeutic intervention for internet gaming disorder. Psychiatry Res. (2022) 314:114633. doi: 10.1016/j.psychres.2022.114633, PMID: 35671563

[25] Wölfling K Beutel ME Dreier M Müller KW. Treatment outcomes in patients with internet addiction: a clinical pilot study on the effects of a cognitive-behavioral therapy program. Biomed Res Int. (2014) 2014:425924. doi: 10.1155/2014/425924 25097858 PMC4100341

[26] Wölfling K Müller KW Dreier M Ruckes C Deuster O Batra A. Efficacy of short-term treatment of internet and computer game addiction: a randomized clinical trial. JAMA Psychiat. (2019) 76:1018–25. doi: 10.1001/jamapsychiatry.2019.1676, PMID: 31290948 PMC6624826

[27] Young KS. Cognitive behavior therapy with internet addicts: treatment outcomes and implications. Cyberpsychol Behav. (2007) 10:671–9. doi: 10.1089/cpb.2007.9971, PMID: 17927535

[28] Young KS. Treatment outcomes using CBT-IA with internet-addicted patients. J Behav Addict. (2013) 2:209–15. doi: 10.1556/JBA.2.2013.4.3, PMID: 25215202 PMC4154573

[29] André F Einarsson I Dahlström E Niklasson K Håkansson A Claesdotter-Knutsson E. Cognitive behavioral treatment for disordered gaming and problem gambling in adolescents: a pilot feasibility study. Ups J Med Sci. (2022) 8:127. doi: 10.48101/ujms.v127.8693, PMID: 35991463 PMC9383045

[30] Du YS Jiang W Vance A. Longer term effect of randomized, controlled group cognitive behavioural therapy for internet addiction in adolescent students in Shanghai. Aust N Z J Psychiatry. (2010) 44:129–34. doi: 10.3109/00048670903282725, PMID: 20113301

[31] Li H Wang S. The role of cognitive distortion in online game addiction among Chinese adolescents. Child Youth Serv Rev. (2013) 35:1468–75. doi: 10.1016/j.childyouth.2013.05.021

[32] Torres-Rodríguez A Griffiths MD Carbonell X Oberst U. Treatment efficacy of a specialized psychotherapy program for internet gaming disorder. J Behav Addict. (2018) 7:939–52. doi: 10.1556/2006.7.2018.111, PMID: 30427213 PMC6376389

[33] Yao Y-W Chen P-R Li C-SR Hare TA Li S Zhang J-T. Combined reality therapy and mindfulness meditation decrease intertemporal decisional impulsivity in young adults with internet gaming disorder. Comput Hum Behav. (2017) 68:210–6. doi: 10.1016/j.chb.2016.11.038

[34] Zhang JT Yao YW Potenza MN Xia CC Lan J Liu L. Effects of craving behavioral intervention on neural substrates of cue-induced craving in internet gaming disorder. Neuroimage Clin. (2016) 12:591–9. doi: 10.1016/j.nicl.2016.09.004, PMID: 27699148 PMC5035334

[35] Brand M Rumpf H-J Demetrovics Z King DL Potenza MN Wegmann E. Gaming disorder is a disorder due to addictive behaviors: evidence from behavioral and neuroscientific studies addressing Cue reactivity and craving, executive functions, and decision-making. Curr Addict Rep. (2019) 6:296–302. doi: 10.1007/s40429-019-00258-y

[36] Jäger S Müller KW Ruckes C Wittig T Batra A Musalek M. Effects of a manualized short-term treatment of internet and computer game addiction (STICA): study protocol for a randomized controlled trial. Trials. (2012) 13:43–3. doi: 10.1186/1745-6215-13-43, PMID: 22540330 PMC3418190

[37] Pape M Geisler BL Cornelsen L Bottel L Te Wildt BT Dreier M. A short-term manual for webcam-based telemedicine treatment of internet use disorders. Front Psych. (2023) 14:1053930. doi: 10.3389/fpsyt.2023.1053930, PMID: 36911137 PMC9995520

[38] Zajac K Ginley MK Chang R Petry NM. Treatments for internet gaming disorder and internet addiction: a systematic review. Psychol Addict Behav. (2017) 31:979–94. doi: 10.1037/adb0000315, PMID: 28921996 PMC5714660

[39] Magill M Ray L Kiluk B Hoadley A Bernstein M Tonigan JS. A Meta-analysis of cognitive-behavioral therapy for alcohol or other drug use disorders: treatment efficacy by contrast condition. J Consult Clin Psychol. (2019) 87:1093–105. doi: 10.1037/ccp0000447, PMID: 31599606 PMC6856400

[40] Magill M Ray LA. Cognitive-behavioral treatment with adult alcohol and illicit drug users: a meta-analysis of randomized controlled trials. J Stud Alcohol Drugs. (2009) 70:516–27. doi: 10.15288/jsad.2009.70.516, PMID: 19515291 PMC2696292

[41] Ray LA Meredith LR Kiluk BD Walthers J Carroll KM Magill M. Combined pharmacotherapy and cognitive behavioral therapy for adults with alcohol or substance use disorders: a systematic review and Meta-analysis. JAMA Netw Open. (2020) 3:e208279. doi: 10.1001/jamanetworkopen.2020.8279, PMID: 32558914 PMC7305524

[42] Walters GD. Behavioral self-control training for problem drinkers: a meta-analysis of randomized control studies. Behav Ther. (2000) 31:135–49. doi: 10.1016/S0005-7894(00)80008-8

[43] Miller WR. Motivational interviewing preparing people for change. New York: Guilford Publications, Inc (2002).

[44] Bischof G Bischof A Rumpf HJ. Motivational interviewing: an evidence-based approach for use in medical practice. Dtsch Arztebl Int. (2021) 118:109–15. doi: 10.3238/arztebl.m2021.0014, PMID: 33835006 PMC8200683

[45] Rubak S Sandbaek A Lauritzen T Christensen B. Motivational interviewing: a systematic review and meta-analysis. Br J Gen Pract. (2005) 55:305–12. 15826439 PMC1463134

[46] Schmidt H Brandt D Meyer C Bischof A Bischof G Trachte A. Motivational brief interventions for adolescents and young adults with internet use disorders: a randomized-controlled trial. J Behav Addict. (2022) 11:754–65. doi: 10.1556/2006.2022.00049, PMID: 36112487 PMC9872541

[47] Beranuy M Carbonell X Griffiths MD. A qualitative analysis of online gaming addicts in treatment. Int J Ment Heal Addict. (2013) 11:149–61. doi: 10.1007/s11469-012-9405-2

[48] Vadlin S Åslund C Hellström C Nilsson KW. Associations between problematic gaming and psychiatric symptoms among adolescents in two samples. Addict Behav. (2015) 61:8–15. doi: 10.1016/j.addbeh.2016.05.001 27203825

[49] Hodgins DC Makarchuk K. Trusting problem gamblers: reliability and validity of self-reported gambling behavior. Psychol Addict Behav. (2003) 17:244–8. doi: 10.1037/0893-164X.17.3.244, PMID: 14498819

[50] Kroenke K Spitzer RL Williams JBW. The PHQ-9: validity of a brief depression severity measure. J Gen Internal Med. (2001) 16:606–13. doi: 10.1046/j.1525-1497.2001.016009606.x, PMID: 11556941 PMC1495268

[51] Spitzer RL Kroenke K Williams JBW Löwe B. A brief measure for assessing generalized anxiety disorder: the GAD-7. Arch Intern Med. (2006) 1960:1092–7. doi: 10.1001/archinte.166.10.1092 16717171

[52] Lindner P Frykheden O Forsström D Andersson E Ljótsson B Hedman E. The Brunnsviken brief quality of life scale (BBQ): development and psychometric evaluation. Cogn Behav Ther. (2016) 45:182–95. doi: 10.1080/16506073.2016.1143526, PMID: 26886248 PMC4867878

[53] Rozental A Forsell E Svensson A Forsström D Andersson G Carlbring P. Psychometric evaluation of the Swedish version of the pure procrastination scale, the irrational procrastination scale, and the susceptibility to temptation scale in a clinical population. BMC Psychol. (2014) 2:54–4. doi: 10.1186/s40359-014-0054-z, PMID: 25566392 PMC4269972

[54] Bergman H Källmén H. Alcohol use among swedes and a psychometric evaluation of the alcohol use disorders identification test. Alcohol Alcohol. (2002) 37:245–51. doi: 10.1093/alcalc/37.3.245, PMID: 12003912

[55] Berman AH Bergman H Palmstierna T Schlyter F. Evaluation of the drug use disorders identification test (DUDIT) in criminal justice and detoxification settings and in a Swedish population sample. Eur Addict Res. (2005) 11:22–31. doi: 10.1159/000081413 15608468

[56] Enders CK. Analyzing longitudinal data with missing values. Rehabil Psychol. (2011) 56:267–88. doi: 10.1037/a0025579 21967118

[57] Gueorguieva R Krystal JH. Move over ANOVA: progress in analyzing repeated-measures data and its reflection in papers published in the archives of general psychiatry. Arch Gen Psychiatry. (2004) 61:310–7. doi: 10.1001/archpsyc.61.3.310 14993119

[58] Cohen J. Statistical power analysis for the behavioral sciences. Hillsdale: L. Erlbaum Associates (1988).

[59] Feingold A. Effect sizes for growth-modeling analysis for controlled clinical trials in the same metric as for classical analysis. Psychol Methods. (2009) 14:43–53. doi: 10.1037/a0014699, PMID: 19271847 PMC2712654

[60] Wells JE Browne MO Aguilar-Gaxiola S Al-Hamzawi A Alonso J Angermeyer MC. Drop out from out-patient mental healthcare in the World Health Organization's world Menta health survey initiative. Br J Psychiatry. (2013) 202:42–9. doi: 10.1192/bjp.bp.112.113134 23174514

[61] Osika Friberg I Krantz G Määttä S Järbrink K. Sex differences in health care consumption in Sweden: a register-based cross-sectional study. Scand J Public Health. (2016) 44:264–73. doi: 10.1177/1403494815618843, PMID: 26647097

[62] Škařupová K Blinka L Ťápal A. Gaming under the influence: an exploratory study. J Behav Addict. (2018) 7:493–8. doi: 10.1556/2006.7.2018.27, PMID: 29788755 PMC6174600

[63] Walther B Morgenstern M Hanewinkel. Co-occurrence of addictive behaviours: personality factors related to substance use, gambling and computer gaming. Eur Addict Res. (2012) 18:167–174. doi: 10.1159/000335662 [Epub ahead of print]., PMID: 22398819

[64] Turel O Bechara A. Little video-gaming in adolescents can be protective, but too much is associated with increased substance use. Subst Use Misuse. (2019) 54:384–395. doi: 10.1080/10826084.2018.1496455 [Epub ahead of print]., PMID: 30654698

[65] André F Håkansson A Claesdotter-Knutsson E. Gaming, substance use and distress within a cohort of online gamblers. J Public Health Res. (2021) 11:3434. doi: 10.4081/jphr.2021.3434, PMID: 35586184 PMC8847953

[66] Krossbakken E Pallesen S Mentzoni RA King DL Molde H Finserås TR. A cross-lagged study of developmental trajectories of video game engagement, addiction, and mental health. Front Psychol. (2018) 9:2239. doi: 10.3389/fpsyg.2018.02239, PMID: 30519203 PMC6258776

[67] Erevik EK Torsheim T Andreassen CS Krossbakken E Vedaa Ø Pallesen S. The associations between low-level gaming, high-level gaming and problematic alcohol use. Addict Behav Rep. (2019) 10:100186. doi: 10.1016/j.abrep.2019.100186, PMID: 31193377 PMC6527943

[68] Andersson Y Ungar OM. (2021). *The Swedish media council*. Available at: https://www.statensmedierad.se/rapporter-och-analyser/material-rapporter-och-analyser/ungar--medier-2021 (Accessed May 15, 2023).

[69] King DL Chamberlain SR Carragher N Billieux J Stein D Mueller K. Screening and assessment tools for gaming disorder: a comprehensive systematic review. Clin Psychol Rev. (2020) 77:101831. doi: 10.1016/j.cpr.2020.101831, PMID: 32143109

[70] Slack JD Delfabbro P King DL. Toward a delineation of the differences between high engagement and problem gaming. Addict Behav Rep. (2022) 16:100462. doi: 10.1016/j.abrep.2022.100462, PMID: 36247099 PMC9554823

[71] Wartberg L Bröning S Lindenberg K. Problematic gaming in youth and its association with different dimensions of quality of life. Z Kinder Jugendpsychiatr Psychother. (2021) 50:9–15. doi: 10.1024/1422-4917/a000810, PMID: 34110245

[72] Fazeli S Mohammadi Zeidi I Lin CY Namdar P Griffiths MD Ahorsu DK. Depression, anxiety, and stress mediate the associations between internet gaming disorder, insomnia, and quality of life during the COVID-19 outbreak. Addict Behav Rep. (2020) 12:100307. doi: 10.1016/j.abrep.2020.100307, PMID: 33110934 PMC7581367

[73] Yeh YC Wang PW Huang MF Lin PC Chen CS Ko CH. The procrastination of internet gaming disorder in young adults: the clinical severity. Psychiatry Res. (2017) 254:258–62. doi: 10.1016/j.psychres.2017.04.055, PMID: 28482194

[74] Lindenberg K Kindt S Szász-Janocha C. Effectiveness of cognitive behavioral therapy-based intervention in preventing gaming disorder and unspecified internet use disorder in adolescents: a cluster randomized clinical trial. JAMA Netw Open. (2022) 5:e2148995. doi: 10.1001/jamanetworkopen.2021.48995, PMID: 35179587 PMC8857686

[75] Wartberg L Lindenberg K. Predictors of spontaneous remission of problematic internet use in adolescence: a one-year follow-up study. Int J Environ Res Public Health. (2020) 17:448. doi: 10.3390/ijerph17020448, PMID: 31936677 PMC7014287

[76] Rothmund T Klimmt C Gollwitzer M. Low temporal stability of excessive video game use in German adolescents. J Media Psychol. (2018) 30:53–65. doi: 10.1027/1864-1105/a000177

